# Purtscher’s retinopathy after intramedullary nailing of a femoral shaft fracture in a 20-year old healthy female – report of a rare case and review of the literature

**DOI:** 10.1186/1471-2474-15-42

**Published:** 2014-02-19

**Authors:** Reinhold Ortmaier, Herbert Resch, Caroline Stieböck, Ottokar Stundner, Eva Maria Arlt

**Affiliations:** 1 University Clinic for Trauma Surgery and Sports Injuries, Müllner Hauptstraße 48, Salzburg A-5020, Austria; 2 University Clinic for Ophthalmology, Müllner Hauptstraße 48, Salzburg A-5020, Austria; 3 University Clinic for Anesthesiology, Müllner Hauptstraße 48, Salzburg A-5020, Austria

## Abstract

**Background:**

Purtscher’s retinopathy is a sight threatening, occlusive microvasculopathy associated with trauma, it is rarely reported after long bone fractures.

**Case presentation:**

A 20-year-old female sustained a femoral shaft fracture (AO 32-A2.3) in a ski accident colliding with a snowgun and was treated with intramedullary nailing one hour after the accident. 14 hours after surgery the patient complained of loss of vision in both eyes and was therefore referred to a neurologist, furthermore an MRI scan of the brain was performed. Neither showed any pathological findings. The patient was finally transferred to an ophthalmology department. After slit lamp examination and funduscopy Purtscher’s retinopathy was diagnosed. Treatment was started right after diagnosis and 5 days after the onset of symptoms. The patient was administered intravenous haemo-rheologic therapy for five days as well as low molecular heparine in therapeutic dose and Vasonit® 400 mg bid orally.

At follow-up 4 weeks and 6 months later visual acuity had improved after 4 weeks before that exam. At final follow-up the symptoms had almost resolved completely and uncorrected visual acuity (UCVA) and best corrected visual acuity had improved from originally 0.25 decimal in both eyes to 0.8 decimal UCVA and BCVA in both eyes.

**Conclusions:**

Patients suffering from perioperative loss of vision have to be referred for ophthalmological and neurological assessment as soon as possible. History of trauma and visual loss can point to the diagnosis of Purtscher’s retinopathy.

## Background

Purtscher’s retinopathy, first described in 1910 by Otmar Purtscher, is an occlusive microvasculopathy associated with trauma [1]. Purtscher described special retinal findings in a man who fell from a tree, sustained severe head injury and suffered from temporary loss of vision. The retinal findings in this patient were retinal hemorrhage and the by Purtscher so called ‘Flecken’ at the posterior pole in both eyes. Purtscher connected these pathological changes with extravasation of lymph from retinal vessels secondary to high intracranial pressure after head injury. Purtscher’s retinopathy is described generally in patients after trauma, e.g. head injury or thoracic compression, whereas the term Purtscher-like-retinopathies refers to patients with this condition but without history of trauma but severe disease as e.g. pancreatitis, renal failure, [2-7]. Incidence of Purtscher and Purtscher-like-retinopathies is estimated to be 0.24 per million per year but might be underestimated due to only few patients presenting to an ophthalmologist with these symptoms [4,7]. Bilateral onset is present in up to 60% [4,7].

Purtscher’s and Purtscher-like-retinopathies are clinical diagnoses built on sudden loss of vision of variable severity and retinal changes like intraretinal hemorrhage, cotton wool spots and Purtscher flecken [2,4]. Flecken are assumed to be the result of the trauma associated occlusion of precapillary arterioles with fat or bone marrow emboli or leukoaggregation. They appear in a polygonal shape in the deep retinal layers and are considered pathognomonic but can only be found in about 50% [2,4,8].

## Case presentation

A 20-year-old fit and healthy female was admitted to the emergency department after collision with a snowgun when skiing. The patient sustained a femoral shaft fracture (AO 32-A2.3) and was treated with intramedullary nailing one hour after the accident (Expert R/AFN, 10/400; DePuy-Synthes, Johnson & Johnson, Warsaw, IN). Due to narrow intramedullary canal, SynReam® (DePuy-Synthes, Johnson & Johnson, Warsaw, IN) was used to enlarge the intramedullary canal diameter up to 11,5 mm to ensure nail insertion. Operation time was 32 minutes, no complications occurred. 14 hours after surgery the patient complained about vision loss in both eyes. She couldn’t read the text messages on her cellphone any longer and reported feeling uncomfortable, dizzy and weak. No other visual or neurological symptoms were present at this point. Blood pressure was relatively low 80/40 mmHg and was 100/50 mmHG after infusion of 250 ml Voluven® (HES 130/0,4) 6% and 500 ml Ringer’s solution, but visual symptoms persisted without improvement. Laboratory findings included elevated leukocytes 18.3 × 10^3^/μl (normal range 4.3-10.0) and LDH 245 U/l (normal range 120–240). Subsequently the patient was referred to a neurologist for neurologic examination and MRI scans of the brain were ordered. However, no pathologic finding, neither in the neurologic examination nor in the MRI scans were detected. The patient was eventually referred to an ophthalmology department, where Purtscher’s retinopathy was diagnosed after slit lamp examination and funduscopy.

Ophthalmological findings and visual outcome:

At first presentation uncorrected visual acuity (UCVA) and best corrected visual acuity (BCVA) were 0.25 decimal in both eyes. A computer perimetry (Humphrey 30:2) could only be performed for the right eye due to the patient’s general malaise after femoral surgery. The field did not show any specific patterns or findings.

Pupils were equal, round and reactive to light, no relative afferent pupillary defect was detected.

**Figure 1:**
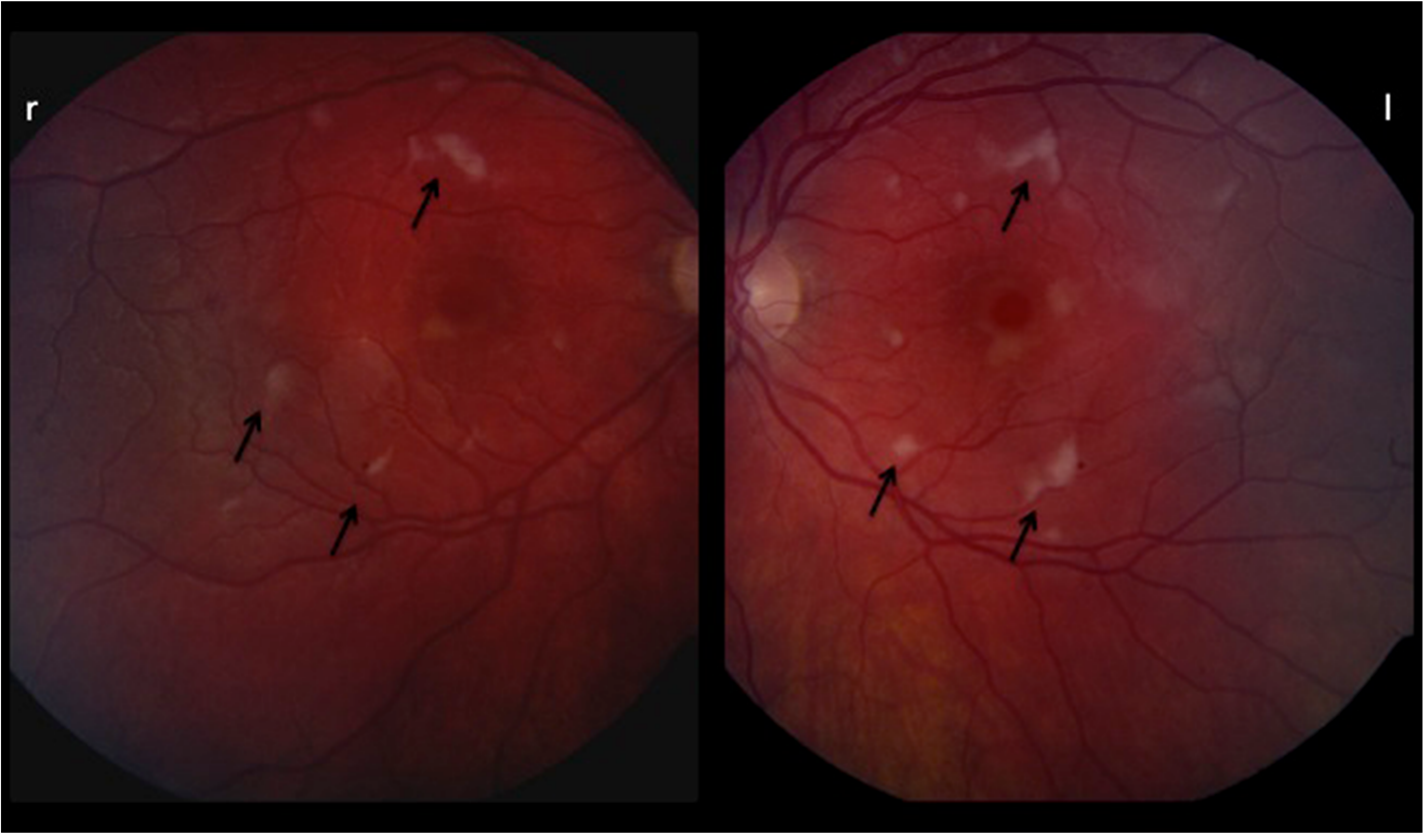
Shows Fundus images of Purtscher's showing Purtscher Flecken.

**Figure 2:**
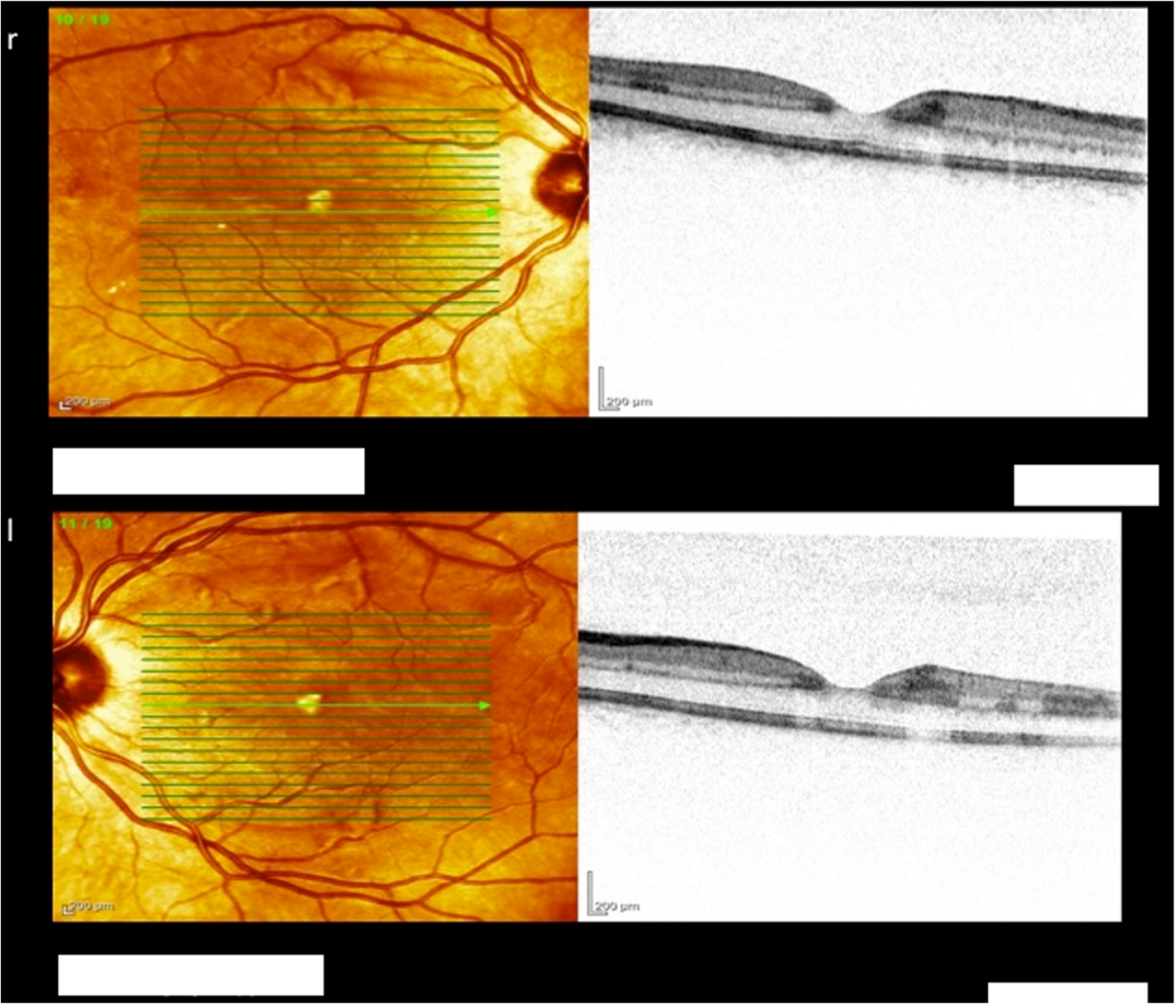
Shows OCT findings in Purtscher's with hyperintense lesions within the retinal nerve fibre layer.

Slit lamp examination showed normal findings in the anterior segment. At fundoscopy cotton wool spots and Purtscher flecken (black arrows, Figure 1) were revealed along the posterior pole in both eyes as well a small intraretinal hemorrhage in the periphery (Figure 1). Additionally to funduscopy an optical coherence tomography (OCT) was performed in both eyes. The findings (Figure 2) showed hyperintense lesions within the retinal nerve fibre layer due to microinfarctions. Summing up these findings Purtscher’s retinopathy was diagnosed.

The patient was administered intravenous haemo-rheologic therapy for five days (Haes® and Trental® in increasing dosage, i.e. 250 ml Haes and 100 mg Trental first day, 250 ml Haes and 200 mg Trental second day, 300 ml Haes and 300 mg Trental days 3, 4 and 5) as well as low molecular heparine in therapeutic dose and Vasonit® 400 mg bid orally.

No particular monitoring regarding the clotting profile was undertaken since there is no evidence based therapy for Purtscher’s and initiated treatment was the sole attempt to improve blood flow in the affected retinal vessels (7).

The patient had follow-up exams after 4 weeks and 6 months. At final follow-up the symptoms had almost resolved completely and visual acuity improved to 0.8 decimal UCVA and BCVA in both eyes.

Funduscopy at the final follow-up showed no more pathologic finding.

## Conclusion

Purtscher’s retinopathy is a rare but sight-threatening disease occurring after several types of trauma (e.g. head trauma, chest compression, long bone fractures), if there is no history of trauma but systemic disease or autoimmune disease as an underlying cause of Purtscher’s the appropriate term is Purtscher-like-retinopathy (e.g. in pancreatitis, renal failure or autoimmune disease) [1-8]. No randomized controlled trials have been published dealing with the treatment of Purtscher’s retinopathy but case reports and case series as well as reviews. Agrawal et al. published a prospective observational study of cases between April 2004 and March 2005, which were reported through the British Ophthalmic Surveillance Unit (BOSU) active reporting scheme [4,7]. An estimated annual incidence for Purtscher’s retinopathy of 0.24 per million per year was found. Although response rate of ophthalmologists was high, this data counts only for the UK and probably is underestimated due to patients who did not present to an ophthalmologist or to ophthalmology departments due to mild symptoms [4,7]. Holló G et al. suspect a much higher incidence of Purtscher’s disease because of impossibility to diagnose patients in intensive care units who are in terminal or severe status [9].

The exact pathogenesis of Purtscher’s and Purtscher-like retinpathy is not completely understood. Purtscher himself postulated extravasation of lymph from retinal veins due to high intracranial pressure after head injury [1]. Since then, other pathomechanisms were described such as venous dilatation due to high intrathoracic pressure after chest compression, vasculitis secondary to lipase release after acute pancreatitis and vascular occlusion due to emboli resulting from air, fat, leukocyte aggregates, complement aggregation, platelets and fibrin [2,10-12].

In patients with long bone fractures – as in our patient - fat embolism obviously seems to be the underlying cause.

Chuang et al. examined 100 patients with long bone fractures and found Cotton-wool spots and retinal hemorrhages in 4%, but only 1% of the patients were symptomatic [12]. Signs of Purtscher’s retinopathy might be found in visually asymptomatic patients with long bone fractures without clinical symptoms [12,13].

Typical clinical symptoms are sudden vision loss of variable severity and in some cases visual field loss. However, also asymptomatic cases were described [2].

In our case, the onset of symptoms was 15 hours after the initial trauma and 14 hours after surgery and a conclusion whether trauma or surgery caused Purtscher’s cannot be drawn due to the very narrow time interval in-between. In Purtscher’s retinopathy onset of visual impairment is usually 24–48 hours after the initial trauma [2]. However, fat embolism syndrome is associated with intramedullary nailing and more likely after reaming of the intramedullary canal [14]. Although release of fat emboli from the intramedullary canal in the venous system seems a common condition after a fracture, reported fat embolism syndrome is a rare condition [12,14]. Generally speaking about patients suffering from vision loss perioperatively one has to take several causes, depending on the surgery and accompanying factors into account (Table 1) and have the patient referred to an ophthalmologist as soon as possible to make the correct diagnosis. However, loss of acuity or visual field, or both in either one eye or both eyes within one day after trauma should remind one of Purtscher retinopathy.

**Table 1:** **Perioperative loss of vision in nonocular surgery – common differential diagnoses**[15-17]

**Site of injury/location of the lesion causing visual problems**	**Specific location**	**Underlying causes**
External ocular injury	Corneal abrasion, corneal exposure	Perioperative exposure of cornea or microtrauma
Retina (retinal ischemia)	Central retinal artery occlusion/occlusion of retinal arterial branch either ischemic or embolic	Occurs mainly in cardiac/vascular surgeries (emboli), but also in spinal surgery due to prone positioning (external compression of the globe), very rarely other forms of surgery (e.g. orthopedic surgery)
	Purtscher’s can be regarded as a special form of this entity	
Ischemic optic neuropathy	Anterior ischemic optic neuropathy, posterior optic neuropthay	Most common site of permanent injury, most often in spinal surgery (prone position), bilateral involvement in most cases
Lesion of retrochiasmal visual pathways	Either homonymous hemianopia (unilateral) or cerebral/cortical visual impairment (bilateral)	Most common mechanism: embolic cerebral infarction (posterior cerebral arteries). Mainly in cardiac surgeries, resection of head and neck tumors

Treatment strategies in Purtscher’s range from watchful waiting to high-dose steroid administration. Until now there are no evidence based treatment guidelines and especially the use of corticosteroids remains unclear. Case reports and series did not show statistically significant improvement in outcome, but this data due to its limitations cannot be taken fully into account and larger prospective cohort studies would be needed [2,4,7,18,19].

In conclusion, Purtscher’s retinopathy is a rare but sight threatening condition and often seen in trauma patients. Associated retinal pathologies often persist more than one month and include Purtscher flecken, cotton wool spots and retinal haemorrhage. A trauma patient with long bone fracture, head injury or chest compression should be evaluated with special regard to visual symptoms and in case of these be referred to an ophthalmologist.

### Consent

Written informed consent was obtained from the patient for publication of this Case report and any accompanying images. A copy of the written consent is available for review by the Editor of this journal

## Competing interests

The authors declare that they have no competing interests.

## Authors’ contributions

OR drafted the manuscript. AE made substantial contributions to the conception of the manuscript. SC accounted for ophthalmic details. HR gave final approval of the version to be published. All authors read and approved the final manuscript.

## Pre-publication history

The pre-publication history for this paper can be accessed here:

http://www.biomedcentral.com/1471-2474/15/42/prepub
